# Effect of socioeconomic status on the healthcare-seeking behavior of migrant workers in China

**DOI:** 10.1371/journal.pone.0237867

**Published:** 2020-08-19

**Authors:** Xuefeng Li, Li Deng, Han Yang, Hui Wang

**Affiliations:** 1 West Center for Economic Research, Southwestern University of Finance and Economics, Chengdu, Sichuan Province, China; 2 School of Finance, Southwestern University of Finance and Economics, Chengdu, Sichuan Province, China; 3 School of Accounting, Southwestern University of Finance and Economics, Chengdu, Sichuan Province, China; Ningbo University, CHINA

## Abstract

In recent years, China has made great efforts to resolve the health inequality caused by household registration restrictions, and the unequal allotment of health services faced by migrant workers has been effectively alleviated. However, inequality in health services may exist not only between migrant workers and local citizens but also among migrant workers. Thus, the unbalanced utilization of health services among migrant workers deserves attention. Using data from the 2017 China Migrants Dynamic Survey (CMDS), we examined the relationship between socioeconomic status (SES) and healthcare-seeking behavior through multivariate regression analysis. Then, from the perspective of SES, this study divided migrant workers into different groups to explore the characteristics of healthcare-seeking behavior in different groups. The results showed that SES had a significant relationship with healthcare-seeking behavior. Those with high SES were more likely to use high-quality health services. By subdividing the category of migrant workers, we found that the utilization of health services among migrant workers was unbalanced. Education and income had significant gradients in multiple measures of healthcare-seeking behavior, while occupation had no significant difference in the behavior. Migrant workers with higher income and education were more likely to use high-quality health services. Especially for migrant workers who had high incomes (above 15,000 CNY) or whose educational backgrounds were graduate level or above, their utilization of health resources was significantly higher than that of other groups. When designing particular policies to improve the healthcare-seeking behavior of different SES migrant workers, we should pay attention to the low-education groups and low-income groups. Policymakers can reduce the current health inequality of migrant workers by strengthening health education and increasing medical subsidies to achieve health equality among migrant workers and between migrant workers and local citizens.

## Introduction

Migrant workers are the labor force that does not change their household registration but works as temporary residents in non-household locations [1]. The household registration system is a kind of population registration management system. In China, citizens are registered according to the administrative region of their birthplace. In 2018, China had 288.36 million migrant workers, accounting for 20.7% of the total population [2]. Although migrant workers have made significant contributions to urban development, they have a difficult time receiving good urban socioeconomic welfare due to the restrictions of the household registration system [3–5]. This difficulty is no exception in the field of health services [6, 7]. As a vulnerable group, migrant workers live at the “edge of the city” and thus lack necessary health protection, which affects the city’s public health. Based on protecting the fundamental human rights of migrant workers and urban public health services, the Chinese government has formulated a series of policies, including the popularization of basic social insurance, to improve the availability and convenience of health services for migrant workers [8, 9].

After years of effort, the unequal allotment of health services faced by migrant workers due to household registration has been effectively alleviated [10–12]. However, the utilization of health services among migrant workers compared with local citizens is still insufficient [13, 14]. Research has shown that the increase in migrant workers’ mobility was related to poor health status and negative healthcare-seeking behavior [15]. To explain this phenomenon, a large number of scholars have conducted empirical research on the factors affecting the healthcare-seeking behavior of migrant workers in China [16–18]. In addition, research on the healthcare-seeking behaviors of migrant workers in other developing countries has also provided useful references for explaining this phenomenon [19–23]. Among the numerous factors that might affect the behavior, income, as a representative variable to measure socioeconomic status (SES), has been widely implicated.

Generally, SES includes prestige, power, and economic welfare [24], which is a comprehensive measure of an individual’s economic and sociological standing. Numerous studies have regarded income, education, and occupation as the quantitative indicators to measure SES [25–28]. At present, most of the research on the SES of migrant workers focuses on income, while the emphases on education and occupation are insufficient. Taking into account the differences in the characteristics of migrant workers in China, general description methods (e.g., low-income, low-education, manual workers) are no longer suitable to describe this group. If we consider one or two aspects of SES to study the factors that affect migrant workers’ healthcare-seeking behavior and do not control other variables of SES, the conclusion may be incomplete. The current inequality in health services may exist not only between migrant workers and local citizens but also among migrant workers. The unbalanced utilization of health services among migrant workers deserves attention.

The behavior of seeking healthcare refers to the social action that people take to confirm the existence of diseases and to seek to alleviate the pain of disease when they feel sick or experience symptoms [29]. According to this definition, the healthcare-seeking behavior of migrant workers should include various aspects, such as (a) whether the migrant workers take social action when they feel sick or suffer from specific symptoms; (b) whether the migrant workers go to medical institutions to confirm the existence of disease and seek to alleviate the suffering of the disease; and (c) the characteristics of migrant workers’ choices of medical institutions. In current studies on migrant workers’ healthcare-seeking behavior, only a few variables were selected to measure the behavior. These studies included in-depth analyses of certain aspects of healthcare-seeking behavior [30–33], which had positive effects on the understanding of the behavior. Nevertheless, if we add more variables to measure different aspects of healthcare-seeking treatment behavior, we will attain a more comprehensive understanding of the behavior.

To provide evidence to achieve the equalization of health services between local citizens and migrant workers, we used nationally representative data to explore the impact of SES on the healthcare-seeking behavior of migrant workers. In this study, we selected income level, education level, and occupational status to measure SES comprehensively and explored the following questions: (a) whether SES will affect the attitudes of migrant workers toward treatment for an illness; (b) whether SES will affect the behavior regarding doctor consultation; and (c) whether SES will affect the hospital choice of migrant workers. By studying the above problems, we summarized the characteristics of different SES groups in healthcare-seeking behavior. The rest of this paper is structured as follows. In Section 2, data sources, measures of the variables, descriptive statistics, and estimation methods are presented. Section 3 shows the empirical results. Section 4 provides discussions. Our conclusions are illustrated in the final section.

## Methods

### Data source

This article used data from the 2017 CMDS conducted by the National Health Commission of China. The CMDS is a nationally representative survey covering 32 province-level administrative units in China, sampling the migrant population aged over 15 years and living in the inflow area for more than one month. Probability-proportional-to-size (PPS) was used as the sampling method in the survey. This method is based on the scale of unit size to sample. The sampling process was divided into three steps by the PPS method. The first step was to select the township-level units from the 32 province-level administrative units. In the second step, the village committees were selected from the selected townships. The last step was that the local village committees chose the migrant population to survey. The survey included the essential characteristics of the migrant population, household income, expenditure, health service utilization, and other factors. This paper aimed to analyze the impact of SES on the healthcare-seeking behavior of migrant workers. After eliminating the samples with unemployment, no illness in the past year and missing values, we extracted the migrant workers with diseases in the past year from the total sample, resulting in 60,945 observations.

### Measures

#### Healthcare-seeking behavior

Healthcare-seeking behavior is a kind of social behavior that seeks to alleviate the pain of diseases when the body feels uncomfortable [29], including the treatment of the disease, the choice of medical institutions and medical expenditure, and other actions. We constructed four variables to measure healthcare-seeking behavior: treatment attitude, doctor consultation, medical institution, and hospital choice. Each variable reflected the different stages of healthcare-seeking behavior. The four variables were based on the same question from the CMDS questionnaire: “where did you go first to check on your latest illness?” There were seven responses, namely, A (local primary hospitals), B (local private clinic), C (local comprehensive/specialized hospitals), D (local drugstore), E (returned to hometown), F (other places), and G (no treatment).

Treatment attitude reflected whether the respondent sought medical treatment in the latest illness (1 = responses A-E, 0 = response G). Doctor consultation indicated whether healthcare was sought through doctors. The sample of this variable was selected from responses A-D (1 = responses A-C, 0 = response D). Medical institutions reflected whether the medical institution was a hospital or a private clinic. Private clinics in China are mainly concentrated at the village/community level [34], with inadequate medical facilities and fewer doctors. Compared with private clinics, hospitals have better medical facilities, more doctors, and higher quality of health services. The sample of this variable was selected from responses A-C (1 = response A and response C, 0 = response B). Hospital choice reflected the level of the hospital. In China, the level of primary hospitals is the lowest among all hospitals. The sample of this variable was selected from responses A and C (1 = response C, 0 = response A). Of particular note, since responses E and F were not closely related to the above four variables, it was challenging to include E and F in a particular variable. Moreover, the sample sizes were too small to be discussed separately; both of them were less than 1% of the total sample. Therefore, we have eliminated the samples of responses E and F.

#### Socioeconomic status

We measured SES from the most commonly used dimensions: income, education, and occupation. Income level was measured by monthly household income. The sample was divided into five groups according to the household monthly income: low income (below 3,000 CNY), relatively low income (between 3,000 CNY to 6,000 CNY), middle income (between 6,000 CNY to10,000 CNY), relatively high income (between 10,000 CNY to 15,000 CNY), and high income (above 15,000 CNY). We took low income as a reference. Education level was measured by the highest educational background received by migrant workers and was divided into five levels from low to high: primary school or below, junior high school, high school, college, graduate or above. We took the primary school or below group as the reference group. Migrant workers’ occupation categories were coded according to their jobs. After referring to the Erikson and Goldthorpe and Portocarero occupational classification method, this paper divided occupations into four categories: manual workers, service personnel, self-employed workers, and professionals, taking manual workers as the reference group. This method of division is still widely used in China [35, 36]. Finally, using principal component analysis, income, education, and occupation status were integrated into a composite variable of SES.

#### Control variables

To control for other variables that may affect healthcare-seeking behavior, according to Andersen’s behavior model, we selected control variables from three aspects: predisposing factors, enabling factors, and need factors [37]. Predisposing factors included gender (1 = male, 0 = female), age, living together (the number of people living together in the household), health service publicity (whether the respondent had heard about the national basic public health service project, 1 = yes, 0 = no), and household registration (1 = rural household, 0 = urban household). In China, due to the household registration system, household registration is generally divided into rural households and urban households. Rural households refer to residents who registered in rural areas, while urban households are registered in urban areas. Enabling factors included health record (whether migrant workers established health records, 1 = yes, 0 = no), basic medical insurance (1 = participated in basic medical insurance, 0 = otherwise), and social security (whether migrant workers had a personal social security card, 1 = yes, 0 = no). Health (1 = unable to live independently, 2 = unhealthy but can live independently, 3 = relatively healthy to 4 = very healthy) was the need factor considered in this study.

### Descriptive statistics

Table 1 presents the descriptive statistics of each variable. The results showed that the vast majority of migrant workers would seek medical treatment after their illness, accounting for 82.4%. After choosing medical treatment, 61.5% of them chose to go to medical institutions. Moreover, 70.5% of migrant workers chose hospitals in medical institutions. Among the samples of respondents choosing hospitals, 44.9% chose primary hospitals.

**Table 1:** Descriptive statistics of variables.

Variables	Mean	S.D.	Min	Max	Observations
**Treatment attitude**	0.824	0.380	0.000	1.000	60,945
**Doctor consultation**	0.615	0.487	0.000	1.000	50,242
**Medical institution**	0.705	0.456	0.000	1.000	30,880
**Hospital choice**	0.449	0.497	0.000	1.000	21,757
**Primary school and below**	0.147	0.354	0.000	1.000	60,945
**Junior high school**	0.431	0.495	0.000	1.000	60,945
**High school**	0.222	0.415	0.000	1.000	60,945
**College**	0.117	0.321	0.000	1.000	60,945
**Graduate and above**	0.084	0.277	0.000	1.000	60,945
**Low income**	0.117	0.321	0.000	1.000	60,945
**Relatively low income**	0.401	0.490	0.000	1.000	60,945
**Middle income**	0.347	0.476	0.000	1.000	60,945
**Relatively high income**	0.071	0.256	0.000	1.000	60,945
**High income**	0.065	0.246	0.000	1.000	60,945
**Manual worker**	0.343	0.475	0.000	1.000	60,945
**Service Personnel**	0.257	0.431	0.000	1.000	60,945
**Self-employed**	0.235	0.424	0.000	1.000	60,945
**Professional**	0.176	0.380	0.000	1.000	60,945
**Gender**	0.555	0.497	0.000	1.000	60,945
**Age**	35.633	9.141	18.00	60.00	60,945
**Household registration**	0.867	0.340	0.000	1.000	60,945
**Health**	3.778	0.464	1.000	4.000	60,945
**Living together**	3.206	1.170	1.000	10.000	60,945
**Health service publicity**	0.601	0.490	0.000	1.000	60,945
**Health record**	0.284	0.451	0.000	1.000	60,945
**Basic medical insurance**	0.931	0.254	0.000	1.000	60,945
**Social security card**	0.536	0.499	0.000	1.000	60,945

In terms of the educational background, 43.1% of the migrant workers had junior high school education, and 22.2% had high school education, indicating that they had generally received primary education. Regarding income, only 11.7% of the migrant workers were in the low-income category, suggesting that most of the migrant workers’ income could meet the needs of daily life. Regarding occupation, the proportions of manual workers, service personnel, self-employed workers, and professionals in the sample were 34.3%, 25.7%, 23.5%, and 17.6%, respectively. Thus, the majority of migrant workers were engaged in jobs with low requirements for education and capital endowment.

In the sample, the proportions of males and females were relatively balanced (55.5% were male). The average age was 35.63 years old, and the average health status was healthy, indicating that the sampled migrant workers were generally the middle-aged healthy labor force. A total of 86.7% of them were agricultural households, indicating that most of the samples came from rural areas. The average number of people living together in the household was 3.21, indicating that most migrant workers lived in the form of small families rather than generations. Moreover, 93.1% of the sampled migrant workers participated in basic medical insurance, 60.1% had heard about the national basic public health service project, 53.6% had applied for social security cards, and only 28.4% had established health records. These results showed that the popularization of basic medical insurance for migrant workers in China had made good progress. However, promotion work in other areas of public health services still needed to be improved.

**Fig 1:**
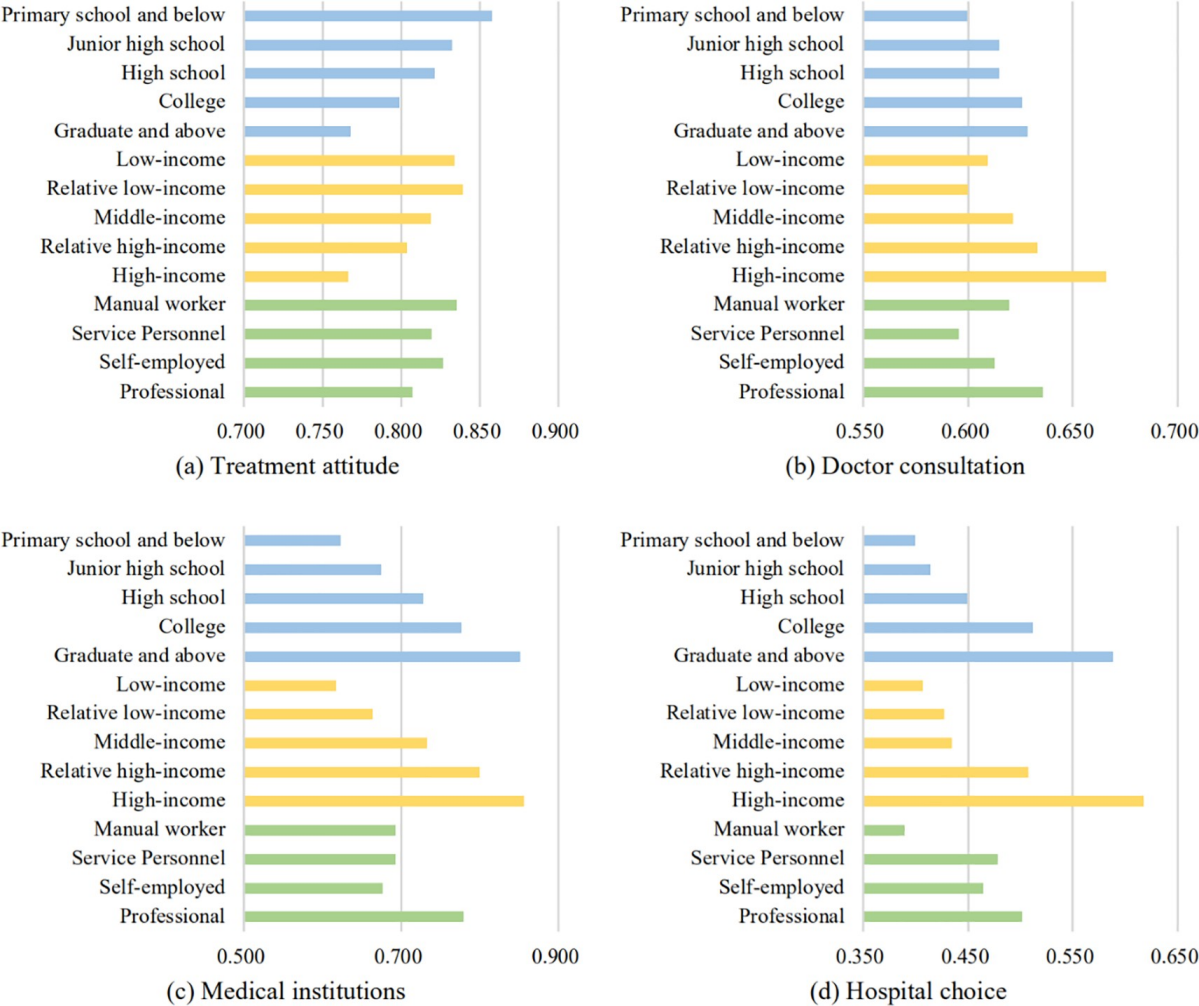
The healthcare-seeking behavior of different groups.

As shown in Fig 1, due to the differences in income, education, and occupation, the migrant workers had apparent differentiation of healthcare-seeking behavior. Income and education performed similarly. With the improvement of these two dimensions, the probability of seeking medical treatment decreased gradually, while doctor consultation, medical institutions, and hospital choice increased gradually. Particularly for migrant workers who had high incomes (above 15,000 CNY) or whose educational backgrounds were graduate or above, their utilization of health resources was significantly higher than those of other groups. In terms of occupation category, the manual worker had the best treatment attitude, but the probability of choosing comprehensive/specialized hospitals for medical treatment was the lowest. The probabilities of choosing hospitals by manual workers, service personnel, and self-employed workers were close, while the probability of that was far higher among professionals than among the other three profession groups.

### Estimation method

After controlling for the essential characteristics of migrant workers, we first examined the relationship between SES and healthcare-seeking behavior through multivariate expression analysis. Due to differences in education, income, and occupation, there might be behavior differences among migrant workers. This study divided migrant workers into different groups from the perspective of SES and discussed the healthcare-seeking behavior of different groups. The first step was to explore whether different groups of migrant workers chose medical services after their illness through two variables: treatment attitude and doctor consultation. In the second step, the medical institution and hospital choice were used to explore the utilization of health services by different groups.

Since the dependent variables in this paper were binary, heteroscedasticity and non-normality might appear when using a linear regression model. Therefore, we applied the Probit model for regression to better control these problems [38]. The model was as follows:

$$Y=\alpha +\beta SES+\sum_{j}\gamma_{j}C_{j}+\varepsilon$$

In this model, *Y* denoted healthcare-seeking behavior, *SES* represented socioeconomic status, *C* represented control variables, and *ε* denoted the error term. We used STATA 13.1 for all statistical analyses.

## Results

Table 2 presents the effect of SES on healthcare-seeking behavior by Probit models among the migrant workers in China. SES was negatively correlated with treatment attitude (β = -0.047, p<0.01) and positively correlated with doctor consultation (β = 0.013, p<0.01), medical institution (β = 0.157, p<0.05), and hospital choice (β = 0.165, p<0.01). These results showed that those with high SES were less likely to seek health services as their first choice after illness. However, once choosing the behavior of seeking healthcare, SES had significant positive effects on doctor consultation, medical institutions, and hospital choice.

**Table 2:** Effect of SES on healthcare-seeking behavior.

	Treatment attitude	Doctor consultation	Medical institution	Hospital choice
**SES**	-0.047^***^	0.013^**^	0.157^***^	0.165^***^
	(0.007)	(0.007)	(0.010)	(0.010)
**Male**	-0.052^***^	-0.019^*^	-0.024	-0.064^***^
	(0.012)	(0.012)	(0.016)	(0.018)
**Age**	0.002^**^	-0.005^***^	0.002^*^	-0.001
	(0.001)	(0.001)	(0.001)	(0.001)
**Household registration**	0.091^***^	0.026	-0.150^***^	-0.054^**^
	(0.019)	(0.019)	(0.028)	(0.028)
**Health**	-0.117^***^	-0.168^***^	-0.085^***^	-0.279^***^
	(0.014)	(0.013)	(0.017)	(0.019)
**Living together**	0.017^***^	0.021^***^	-0.038^***^	-0.002
	(0.005)	(0.005)	(0.007)	(0.008)
**Health service publicity**	0.071^***^	0.078^***^	0.119^***^	-0.152^***^
	(0.014)	(0.013)	(0.018)	(0.020)
**Health record**	0.080^***^	0.072^***^	0.076^***^	-0.189^***^
	(0.016)	(0.014)	(0.020)	(0.021)
**Basic medical insurance**	0.079^***^	0.042^*^	0.064^*^	-0.026
	(0.024)	(0.024)	(0.033)	(0.038)
**Social security card**	-0.010	0.053^***^	0.124^***^	0.102^***^
	(0.014)	(0.013)	(0.018)	(0.020)
**Province level fixed effect**	Yes	Yes	Yes	Yes
**Observations**	60,945	50,242	30,880	21,757

The number in brackets is the standard error.

^*^, ^**^, and ^***^ denote significance at the 10%, 5%, and 1% levels, respectively. All results are robust regression results.

Table 3 shows the effects of SES on treatment attitude and doctor consultation by Probit models among the migrant workers in China. In Model 1, taking primary school and below as the reference, the educational background of each level was negatively related to the treatment attitude, whereas no significant correlation was found for doctor consultation. With the improvement of education, the negative impacts on the treatment attitude increased from junior high school (β = -0.049, p<0.05), high school (β = -0.090, p<0.01), and college (β = -0.141, p<0.01) to graduate and above (β = -0.178, p<0.01). The results of Model 2 showed that, compared with low income, relatively low income had a higher probability of seeking disease treatment (β = 0.044, p<0.05) but a lower probability of doctor consultation (β = -0.045, p<0.05). In contrast, the high-income group had a lower probability of seeking disease treatment (β = -0.079, p<0.05) but a higher probability of doctor consultation (β = 0.072, p<0.05) than low-income groups. Migrant workers in the middle-income and relatively high-income categories had no significant relationships with treatment attitude and doctor consultation. In Model 3, the results indicated that compared with manual workers, service personnel (β = -0.055, p<0.01), self-employed workers (β = -0.096, p<0.01), and professionals (β = -0.033, p<0.1) were less likely to seek health services. Furthermore, when they chose to seek health services, the probabilities of doctor consultation for service personnel (β = -0.071, p<0.01) and self-employed workers (β = -0.037, p<0.05) were still lower than that for manual workers, while professionals had no significant relationship with this behavior.

**Table 3:** Effect of SES on treatment attitude and doctor consultation.

	Treatment attitude				Doctor consultation			
	Model 1	Model 2	Model 3	Model 4	Model 1	Model 2	Model 3	Model 4
**Education (primary school and below as reference)**								
**Junior high school**	-0.049^**^			-0.041^**^	0.016			0.019
	(0.020)			(0.020)	(0.018)			(0.018)
**High school**	-0.090^***^			-0.077^***^	-0.003			-0.002
	(0.023)			(0.023)	(0.021)			(0.021)
**College**	-0.141^***^			-0.133^***^	0.032			0.020
	(0.027)			(0.028)	(0.026)			(0.026)
**Graduate and above**	-0.178^***^			-0.173^***^	0.040			0.008
	(0.030)			(0.032)	(0.029)			(0.031)
**Income (low-income as reference)**								
**Relatively low-income**		0.044^**^		0.054^**^		-0.045^**^		-0.045^**^
		(0.021)		(0.021)		(0.019)		(0.019)
**Middle-income**		0.007		0.029		-0.025		-0.024
		(0.022)		(0.022)		(0.021)		(0.021)
**Relatively high-income**		-0.017		0.025		-0.017		-0.014
		(0.030)		(0.031)		(0.029)		(0.030)
**High-income**		-0.079^**^		-0.015		0.072^**^		0.077^**^
		(0.031)		(0.032)		(0.031)		(0.032)
**Occupation (manual worker as reference)**								
**Service Personnel**			-0.055^***^	-0.044^***^			-0.071^***^	-0.068^***^
			(0.017)	(0.017)			(0.016)	(0.016)
**Self-employed**			-0.096^***^	-0.085^***^			-0.037^**^	-0.028^*^
			(0.017)	(0.017)			(0.016)	(0.016)
**Professional**			-0.033^*^	0.005			0.012	0.017
			(0.019)	(0.019)			(0.019)	(0.018)
**Control variables**	Yes	Yes	Yes	Yes	Yes	Yes	Yes	Yes
**Province level fixed effect**	Yes	Yes	Yes	Yes	Yes	Yes	Yes	Yes
**Observations**	60,945	60,945	60,945	60,945	50,242	50,242	50,242	50,242

The number in brackets is the standard error.

^*^, ^**^, and ^***^ denote significance at the 10%, 5%, and 1% levels, respectively. All results are robust regression results. The constant term is not reported in the table. Model 1–3 takes education level, income level, and occupation status as dependent variables, separately. Model 4 takes education level, income level, and occupation status as dependent variables.

Table 4 shows the effect of SES on medical institution and hospital choice. Through the results of Models 1 and 2, we found that with improvements in education level and income level, the positive effects on the choice of medical institutions and hospitals gradually expanded, indicating that both education and income would increase the probability of migrant workers seeking higher quality health services. Model 3 treated manual workers as a reference. The results showed that compared with manual workers, service personnel (β = 0.070, p<0.01), self-employed workers (β = 0.076, p<0.01), and professionals (β = 0.154, p<0.01) were more willing to seek health services from hospitals. Moreover, service personnel (β = 0.215, p<0.01), self-employed workers (β = 0.222, p<0.01), and professionals (β = 0.229, p<0.01) were more inclined to choose comprehensive/specialized hospitals than manual workers. However, it is worth noting that in Model 4, when education and income were controlled for, the effect of occupation on the probability of choosing hospitals when seeking healthcare was significantly reduced.

**Table 4:** Effect of SES on medical institution and hospital choice.

	Medical institution				Hospital choice			
	Model 1	Model 2	Model 3	Model 4	Model 1	Model 2	Model 3	Model 4
**Education (primary school and below as reference)**								
**Junior high school**	0.126^***^			0.105^***^	0.131^***^			0.091^***^
	(0.024)			(0.024)	(0.029)			(0.029)
**High school**	0.287^***^			0.250^***^	0.224^***^			0.152^***^
	(0.029)			(0.029)	(0.033)			(0.034)
**College**	0.413^***^			0.355^***^	0.364^***^			0.263^***^
	(0.036)			(0.037)	(0.039)			(0.041)
**Graduate and above**	0.568^***^			0.484^***^	0.485^***^			0.347^***^
	(0.044)			(0.046)	(0.044)			(0.047)
**Income (low-income as reference)**								
**Relatively low-income**		0.081^***^		0.065^**^		0.121^***^		0.101^***^
		(0.026)		(0.026)		(0.032)		(0.032)
**Middle-income**		0.171^***^		0.127^***^		0.169^***^		0.123^***^
		(0.027)		(0.028)		(0.033)		(0.033)
**Relatively high-income**		0.328^***^		0.249^***^		0.342^***^		0.254^***^
		(0.041)		(0.041)		(0.043)		(0.044)
**High-income**		0.451^***^		0.358^***^		0.539^***^		0.413^***^
		(0.046)		(0.047)		(0.045)		(0.046)
**Occupation (manual worker as reference)**								
**Service Personnel**			0.070^***^	0.037^*^			0.215^***^	0.185^***^
			(0.022)	(0.022)			(0.024)	(0.025)
**Self-employed**			0.076^***^	0.026			0.222^***^	0.169^***^
			(0.022)	(0.022)			(0.025)	(0.025)
**Professional**			0.154^***^	0.041			0.229^***^	0.134^***^
			(0.025)	(0.027)			(0.026)	(0.027)
**Control variables**	Yes	Yes	Yes	Yes	Yes	Yes	Yes	Yes
**Province level fixed effect**	Yes	Yes	Yes	Yes	Yes	Yes	Yes	Yes
**Observations**	30,880	30,880	30,880	30,880	21,757	21,757	21,757	21,757

The number in brackets is the standard error.

^*^, ^**^, and ^***^ denote significance at the 10%, 5%, and 1% levels, respectively. All results are robust regression results. The constant term is not reported in the table. Model 1–3 takes education level, income level, and occupation status as dependent variables, separately. Model 4 takes education level, income level, and occupation status as dependent variables.

## Discussion

Using nationally representative data in China, our study estimated the association between SES and healthcare-seeking behavior measured by treatment attitude, doctor consultation, medical institution, and hospital choice among migrant workers. Although discrepancies were observed in the relationships between multiple measures of SES and healthcare-seeking behavior, we found some regular conclusions in the empirical results. Our results revealed a significant relationship between SES and healthcare-seeking behavior and a significant gradient in multiple measures of healthcare-seeking behavior by education and income.

SES, a composite variable composed of education, income, and occupation, had significant positive relationships with doctor consultation, medical institutions, and hospital choice among migrant workers. These results showed that, in the utilization of health services, especially high-level health services, high-SES migrant workers had more advantages. Compared with migrant workers with low SES, migrant workers with high SES had a better living environment and more resources [39, 40]. Therefore, considering that medical supplies are classified according to quality level, migrant workers with high SES might be more capable of obtaining better medical services due to their resource advantages. However, SES had a significant negative relationship with treatment attitude. The possible reason for this difference was that the high-SES migrant workers might have better health literacy, indicating the ability to obtain, understand and use information about health and health services [41, 42]. They might have a better understanding of their situation and could determine whether they need health service resources or not.

Migrant workers with higher education levels were less likely to seek medical treatment. This phenomenon might be because highly educated patients are more confident in challenging doctors and do not regard doctors as the preferred solution [43]. Considering the relationship between education and health literacy [44, 45], highly educated migrant workers could make better decisions about whether to seek medical treatment by evaluating their condition. Affected by credible information, relevant knowledge, and experience, those with low education levels might be disadvantaged in making health decisions [46]. Furthermore, education level also had significant positive correlations with the medical institution and hospital choice, and these positive correlations showed gradient increasing trends with the improvement in education level. These results indicated that the highly educated migrant workers were more inclined to choose high-quality health services. Migrant workers with higher education levels might have received health education to a greater extent, which would significantly improve their awareness of health services [47, 48].

Income did not show natural characteristics in treatment attitude and doctor consultation. However, similar to the results of education, income had positive relationships with medical institution and hospital choice, possibly because high-income migrant workers can afford the cost of high-quality health services. The high cost of health services was a significant obstacle for migrant workers to access healthcare [13]. In China, the healthcare processes of hospitals are more complicated than those of private clinics, and the corresponding costs are also high. Although this problem has been alleviated by popularizing basic medical insurance in recent years, migrant workers still have to bear a certain proportion of the cost. Compared with hospitals, private clinics have a lower quality of health services in general [34, 49] and usually adopt a low-price strategy to attract patients [50]. Therefore, low-income people tend to choose private clinics.

Compared with the impact of education and income, the impact of occupation on migrant workers’ healthcare-seeking behavior had no apparent regularity. Service personnel and self-employed workers were less likely to take remedial action and choose healthcare institutions after illness than manual workers, possibly because service personnel and self-employed workers usually have irregular working hours and might not have enough time to seek health services. In the choice of healthcare institutions, service personnel, self-employed workers, and professionals were more inclined to choose comprehensive/specialized hospitals than manual workers, possibly because the former have a higher occupation status than manual workers and can obtain better health resources. However, in terms of the impact on hospital choice, the corresponding coefficients did not increase with an increase in occupation status. Furthermore, by analyzing the results of Models 1–4, we found that after adding education and income as independent variables, the influence of some occupation categories on healthcare-seeking behavior changed from significant to insignificant. Considering the correlations between education, income, and occupation [51], the impact of occupation on healthcare-seeking behavior might be explained by the other two variables.

Currently, low income, low education, and manual labor are no longer synonymous with migrant workers in China. Within the migration worker population, there has been a group differentiation represented by SES. In some specific groups, such as high-income migrant workers, the medical institutions were significantly different from those of other groups. If we treat migrant workers as a group with unified SES characteristics, we will inevitably make mistakes in the analysis of behavior characteristics. According to the results of this study, we propose that when designing particular policies to improve the healthcare-seeking behavior of different SES migrant workers, we should pay attention to the low-education and low-income groups. Furthermore, policy design based on the occupation category may not be a practical choice. Therefore, policymakers can address the current health inequality problems of migrant workers by strengthening health education and increasing medical subsidies to achieve health equality among migrant workers and between migrant workers and local citizens.

Our research had several limitations. First, we chose migrant workers who were ill in the past year as the research object, but due to the restriction of data sources, we did not distinguish the severity of their diseases. Differences in disease severity may have a direct impact on the healthcare-seeking behavior of migrant workers. Although we alleviated this problem by controlling for the health level of migrant workers, it would be better to distinguish the severity of disease in a follow-up study. Second, healthcare-seeking behavior is a general concept, including many aspects of decision-making. In this paper, treatment attitude, doctor consultation, medical institutions, and hospital choice were selected to measure the behavior, but these variables could not cover all aspects of healthcare-seeking behavior. In future studies, more variables should be chosen to improve the measurement of healthcare-seeking behavior, such as treatment options after entering the hospital. Third, the health service policies for migrant workers varied from province to province; thus, the effects of different policies could not be determined. To control the influence of variables at the province level, we added dummy variables of provinces to the analyses. Future studies can further explore the factors that lead to differences in healthcare-seeking behavior among provinces.

## Conclusions

Generally, the differentiation of migrant workers caused by SES had different effects on the characteristics of healthcare-seeking behavior. High-SES migrant workers had more advantages in the utilization of health services, especially high-level health services. At present, the SES of migrant workers in China is characterized by inherent differences, leading to an internal imbalance in the utilization of health services. The healthcare-seeking behaviors of migrant workers with high income and high education were significantly different from those of other groups. To achieve health equality between migrant workers and local citizens, we should also pay attention to the equality of health services among migrant workers. The reform of China’s health system should promote the fairness of health service utilization by formulating reasonable policies to eliminate the above inequalities.

## Supporting information

S1 Dataset (DTA)

## List of abbreviations

CMDS: China Migrants Dynamic Survey
SES: socioeconomic status

## References

[1] Hargreaves S, Rustage K, Nellums LB, McAlpine A, Pocock N, Devakumar D, et al Occupational health outcomes among international migrant workers: a systematic review and meta-analysis. The Lancet Global Health. 2019;7(7):e872–e82. doi: 10.1016/S2214-109X(19)30204-9 31122905 PMC6565984

[2] National Bureau of Statistics of China. 2018 Migrant Workers Monitoring Survey Report. 2018. http://www.stats.gov.cn/tjsj/zxfb/201904/t20190429_1662268.html. Accessed 29 April 2019.

[3] Liu Z. Institution and inequality: the hukou system in China. Journal of Comparative Economics 2005;33:133–57. 10.1016/j.jce.2004.11.001

[4] Wong DFK, Li CY, Song HX. Rural migrant workers in urban China: living a marginalised life. International Journal of Social Welfare 2006;16:32–40. 10.1111/j.1468-2397.2007.00475.x

[5] Afridi F, Li SX, Ren Y. Social identity and inequality: The impact of Chinas hukou system. Journal of Public Economics 2015;123:17–29. 10.1016/j.jpubeco.2014.12.011

[6] Shao C, Meng X, Cui S, Wang J, Li C. Income-related health inequality of migrant workers in China and its decomposition: An analysis based on the 2012 China Labor-force Dynamics Survey data. Journal of the Chinese Medical Association 2016;79:531–7. 10.1016/j.jcma.2016.02.009 27288189

[7] Zhu Y, Hu X, Yang B, Wu G, Wang Z, Xue Z, et al Association between migrant worker experience, limitations on insurance coverage, and hospitalization for schizophrenia in Hunan Province, China. Schizophrenia Research 2018;197:93–7. 10.1016/j.schres.2017.11.026 29195746

[8] Wu J, Yu Z, Wei YD, Yang L. Changing distribution of migrant population and its influencing factors in urban China: Economic transition, public policy, and amenities. Habitat International 2019;94:102063 10.1016/j.habitatint.2019.102063

[9] Zeng H, Yu X, Zhang J. Urban village demolition, migrant workers rental costs and housing choices: Evidence from Hangzhou, China. Cities 2019;94:70–9. 10.1016/j.cities.2019.05.029

[10] Qin X, Pan J, Liu GG. Does participating in health insurance benefit the migrant workers in China? An empirical investigation. China Economic Review 2014;30:263–78. 10.1016/j.chieco.2014.07.009

[11] Hou Z, Zhang D. Health insurance coverage and inpatient services choice among rural-to-urban migrants from a nationwide cross-sectional survey in China: does location matter? The Lancet 2017;390 10.1016/s0140-6736(17)33165-3

[12] Maimaitijiang R, He Q, Wu Y, Bouey JZH, Koné A, Liang Y, et al Assessment of the health status and health service perceptions of international migrants coming to Guangzhou, China, from high-, middle- and low-income countries. Globalization and Health 2019;15 10.1186/s12992-019-0449-y 30683119 PMC6346560

[13] Peng Y, Chang W, Zhou H, Hu H, Liang W. Factors associated with health-seeking behavior among migrant workers in Beijing, China. BMC Health Services Research 2010;10 10.1186/1472-6963-10-69 20298613 PMC2848137

[14] Hao AH, Zhang W, Liu ZF, Xu MJ, Xu N, Liu LP, et al Analysis of basic public health services utilization and influence factors of the floating population in the Pearl River Delta. Chinese Journal of Public Health Management, 2016;32:613–617. (in Chinese).

[15] Li X, Stanton B, Chen X, Hong Y, Fang X, Lin D, et al Health Indicators and Geographic Mobility among Young Rural-to-Urban Migrants in China. World Health & Population 2006;8:5–21. 10.12927/whp.2006.18148 18277098 PMC2249565

[16] Lu C-H, Wang P-X, Lei Y-X, Luo Z-C. Influence of health-related quality of life on health service utilization in Chinese rural-to-urban female migrant workers. Health and Quality of Life Outcomes 2014;12 10.1186/s12955-014-0121-4 25123983 PMC4168158

[17] Zhang J, Lin S, Liang D, Qian Y, Zhang D, Hou Z. Public Health Services Utilization and Its Determinants among Internal Migrants in China: Evidence from a Nationally Representative Survey. International Journal of Environmental Research and Public Health 2017;14:1002 10.3390/ijerph14091002 28862682 PMC5615539

[18] Lu L, Zeng J, Zeng Z. What limits the utilization of health services among china labor force? analysis of inequalities in demographic, socio-economic and health status. International Journal for Equity in Health 2017;16 10.1186/s12939-017-0523-0 28148264 PMC5289053

[19] Seeberg J, Pannarunothai S, Padmawati RS, Trisnantoro L, Barua N, Pandav CS. Treatment seeking and health financing in selected poor urban neighbourhoods in India, Indonesia and Thailand. Social Science & Medicine 2014;102:49–57. 10.1016/j.socscimed.2013.11.039 24565141

[20] Lattof SR. Health insurance and care-seeking behaviours of female migrants in Accra, Ghana. Health Policy and Planning 2018;33:505–15. 10.1093/heapol/czy012 29462305 PMC5894076

[21] Fernandez B. Health inequities faced by Ethiopian migrant domestic workers in Lebanon. Health & Place 2018;50:154–61. 10.1016/j.healthplace.2018.01.008 29454243

[22] Babu B, Sharma Y, Kusuma Y, Sivakami M, Lal D, Marimuthu P, et al Patient experiences and health system responsiveness among internal migrants: A nationwide study in 13 Indian cities. Journal of Healthcare Quality Research 2019;34:167–75. 10.1016/j.jhqr.2019.04.003 31713527

[23] Shriraam V, Srihari R, Gayathri T, Murali L. Active case finding for Tuberculosis among migrant brick kiln workers in South India. Indian Journal of Tuberculosis 2019 10.1016/j.ijtb.2019.09.003 32192615

[24] Conger RD, Conger KJ, Martin MJ. Socioeconomic Status, Family Processes,and Individual Development. Journal of Marriage and Family 2010;72:685–704. 10.1111/j.1741-3737.2010.00725.x 20676350 PMC2910915

[25] Jimenez MP, Wellenius GA, Subramanian S, Buka S, Eaton C, Gilman SE, et al Longitudinal associations of neighborhood socioeconomic status with cardiovascular risk factors: A 46-year follow-up study. Social Science & Medicine 2019;241:112574 10.1016/j.socscimed.2019.112574 31593787 PMC6913883

[26] Chen A, Lakdawalla DN. Healing the poor: The influence of patient socioeconomic status on physician supply responses. Journal of Health Economics 2019;64:43–54. 10.1016/j.jhealeco.2019.02.001 30797112 PMC6481618

[27] Liu T, Zhang X, Jiang Y. Family socioeconomic status and the cognitive competence of very young children from migrant and non-migrant Chinese families: The mediating role of parenting self-efficacy and parental involvement. Early Childhood Research Quarterly 2020;51:229–41. 10.1016/j.ecresq.2019.12.004

[28] Li X, Yang H, Wang H, Jia J. Family socioeconomic status and home-based parental involvement: A mediation analysis of parental attitudes and expectations. Children and Youth Services Review. 2020;116:105111. doi: 10.1016/j.childyouth.2020.105111.

[29] Kasl SV, Cobb S. Some psychological factors associated with illness behavior and selected illnesses. Journal of Chronic Diseases 1964;17:325–45. 10.1016/0021-9681(64)90074-8 14141839

[30] Deng Z, Liu S. Understanding consumer health information-seeking behavior from the perspective of the risk perception attitude framework and social support in mobile social media websites. International Journal of Medical Informatics 2017;105:98–109. 10.1016/j.ijmedinf.2017.05.014 28750916

[31] Liu L, Chen X-L, Ni C-P, Yang P, Huang Y-Q, Liu Z-R, et al Survey on the use of mental health services and help-seeking behaviors in a community population in Northwestern China. Psychiatry Research 2018;262:135–40. 10.1016/j.psychres.2018.02.010 29433108

[32] Li L, Xu W, Wagner AL, Dong X, Yin J, Zhang Y, et al Evaluation of health education interventions on Chinese factory workers’ knowledge, practices, and behaviors related to infectious disease. Journal of Infection and Public Health 2019;12:70–6. 10.1016/j.jiph.2018.09.004 30262191

[33] Armenta A, Sarabia H. Receptionists, doctors, and social workers: Examining undocumented immigrant womens perceptions of health services. Social Science & Medicine 2020;246:112788 10.1016/j.socscimed.2020.112788 31958616

[34] Wang Q, Zhang D, Hou Z. Insurance coverage and socioeconomic differences in patient choice between private and public health care providers in China. Social Science & Medicine 2016;170:124–32. 10.1016/j.socscimed.2016.10.016 27771545

[35] Xie Z, Poon AN, Wu Z, Jian W, Chan KY. Is Occupation a Good Predictor of Self-Rated Health in China? Plos One 2015;10 10.1371/journal.pone.0125274 25951087 PMC4423882

[36] Wang Q, Shen JJ, Sotero M, Li CA, Hou Z. Income, occupation and education: Are they related to smoking behaviors in China? Plos One 2018;13 10.1371/journal.pone.0192571 29420649 PMC5805321

[37] Andersen RM. Revisiting the Behavioral Model and Access to Medical Care: Does it Matter? Journal of Health and Social Behavior 1995;36:1 10.2307/2137284 7738325

[38] Long JS. Regression models for categorical and limited dependent variables. Thousand Oaks (Calif.): Sage Publications; 2011.

[39] Wang L, You Y, Yang C-M. Restrained by resources: The effect of scarcity cues and childhood socioeconomic status (SES) on consumer preference for feasibility. International Journal of Research in Marketing 2020 10.1016/j.ijresmar.2020.01.007

[40] Sheehy-Skeffington J. The effects of low socioeconomic status on decision-making processes. Current Opinion in Psychology 2020;33:183–8. 10.1016/j.copsyc.2019.07.043 31494518

[41] Batterham R, Hawkins M, Collins P, Buchbinder R, Osborne R. Health literacy: applying current concepts to improve health services and reduce health inequalities. Public Health 2016;132:3–12. 10.1016/j.puhe.2016.01.001 26872738

[42] Rydland H. T., Fjær E. L., Eikemo T. A., Huijts T., Bambra C., Wendt C., et al Educational inequalities in mortality amenable to healthcare. A comparison of European healthcare systems. Plos One 2020; 15 10.1371/journal.pone.0234135 32614848 PMC7332057

[43] Smith SK, Dixon A, Trevena L, Nutbeam D, Mccaffery KJ. Exploring patient involvement in healthcare decision making across different education and functional health literacy groups. Social Science & Medicine 2009;69:1805–12. 10.1016/j.socscimed.2009.09.056 19846245

[44] Pandit AU, Tang JW, Bailey SC, Davis TC, Bocchini MV, Persell SD, et al Education, literacy, and health: Mediating effects on hypertension knowledge and control. Patient Education and Counseling 2009;75:381–5. 10.1016/j.pec.2009.04.006 19442477

[45] Lastrucci V., Lorini C., Caini S., & Bonaccorsi G. Health literacy as a mediator of the relationship between socioeconomic status and health: A cross-sectional study in a population-based sample in Florence. Plos One 2019; 14 10.1371/journal.pone.0227007 31869381 PMC6927637

[46] Perez SL, Kravitz RL, Bell RA, Chan MS, Paterniti DA. Characterizing internet health information seeking strategies by socioeconomic status: a mixed methods approach. BMC Medical Informatics and Decision Making 2016;16 10.1186/s12911-016-0344-x 27506607 PMC4979125

[47] Izudi J, Akwang DG, Mccoy SI, Bajunirwe F, Kadengye DT. Effect of health education on birth preparedness and complication readiness on the use of maternal health services: A propensity score-matched analysis. Midwifery 2019;78:78–84. 10.1016/j.midw.2019.08.003 31400596

[48] Barman B, Saha J, Chouhan P. Impact of education on the utilization of maternal health care services: An investigation from National Family Health Survey (2015–16) in India. Children and Youth Services Review 2020;108:104642 10.1016/j.childyouth.2019.104642

[49] Eggleston K, Lu M, Li C, Wang J, Yang Z, Zhang J, et al Comparing public and private hospitals in China: Evidence from Guangdong. BMC Health Services Research 2010;10 10.1186/1472-6963-10-76 20331886 PMC2858143

[50] Liu Y, Berman P, Yip W, Liang H, Meng Q, Qu J, et al Health care in China: The role of non-government providers. Health Policy 2006;77:212–20. 10.1016/j.healthpol.2005.07.002 16112771

[51] Scanlon M, Powell F, Leahy P, Jenkinson H, Byrne O. ‘No one in our family ever went to college’: Parents’ orientations towards their children’s post-secondary education and future occupations. International Journal of Educational Research 2019;93:13–22. 10.1016/j.ijer.2018.09.005

